# Phylogeny of Dictyoptera: Dating the Origin of Cockroaches, Praying Mantises and Termites with Molecular Data and Controlled Fossil Evidence

**DOI:** 10.1371/journal.pone.0130127

**Published:** 2015-07-22

**Authors:** Frédéric Legendre, André Nel, Gavin J. Svenson, Tony Robillard, Roseli Pellens, Philippe Grandcolas

**Affiliations:** 1 Institut de Systématique, Evolution, Biodiversité, ISYEB—UMR 7205 MNHN, CNRS, UPMC, EPHE, Sorbonne Universités, Muséum national d’Histoire naturelle, Département Systématique et Evolution, Paris, France; 2 Department of Invertebrate Zoology, Cleveland Museum of Natural History, Cleveland, Ohio, United States of America; Laboratoire Arago, FRANCE

## Abstract

Understanding the origin and diversification of organisms requires a good phylogenetic estimate of their age and diversification rates. This estimate can be difficult to obtain when samples are limited and fossil records are disputed, as in Dictyoptera. To choose among competing hypotheses of origin for dictyopteran suborders, we root a phylogenetic analysis (~800 taxa, 10 kbp) within a large selection of outgroups and calibrate datings with fossils attributed to lineages with clear synapomorphies. We find the following topology: (mantises, (other cockroaches, (Cryptocercidae, termites)). Our datings suggest that crown-Dictyoptera—and stem-mantises—would date back to the Late Carboniferous (~ 300 Mya), a result compatible with the oldest putative fossil of stem-dictyoptera. Crown-mantises, however, would be much more recent (~ 200 Mya; Triassic/Jurassic boundary). This pattern (i.e., old origin and more recent diversification) suggests a scenario of replacement in carnivory among polyneopterous insects. The most recent common ancestor of (cockroaches + termites) would date back to the Permian (~275 Mya), which contradicts the hypothesis of a Devonian origin of cockroaches. Stem-termites would date back to the Triassic/Jurassic boundary, which refutes a Triassic origin. We suggest directions in extant and extinct species sampling to sharpen this chronological framework and dictyopteran evolutionary studies.

## Introduction

Understanding the origin and diversification of organisms in their environmental context requires a good estimate of their age and diversification rates. This objective is classically achieved through analyses combining morphological and environmental data, molecular phylogenies, and the fossil record [1–3]. These analyses are, however, sometimes inconclusive, especially when the fossil record is disputed, scarce or incomplete (a limitation inherent to fossils) or when molecular phylogenies rely on limited samples [4–6]. Special attention must therefore be paid to improve character and taxon sampling in phylogenies and to evaluate the quality of the fossil record [7,8]. Despite these recommendations, obtaining additional data may be difficult even with a strong sampling effort, especially for fossils for which complete specimens in good state of preservation and phylogenetically relevant are not easily found. To address these limitations and produce a robust analysis, several research strategies designed recently include: integrating the quality of fossil record into the calibration [9,10]; basing dating methods on statistical distributions to account for uncertainties [11]; and nesting the study in a deeper group better-represented in the fossil record and including appropriate outgroups [12–14].

These problems of incomplete or controversial fossil record and molecular phylogenies with limited samples occur in different taxonomic groups. One patent example is Dictyoptera–an insect group including cockroaches, praying mantises and termites, the latter being considered recently as a suborder of Blattodea [15,16]. First, the oldest ‘Dictyoptera-like’ fossils would be useful to date the oldest nodes but these fossils are controversial (see below). Thus, they cannot be readily used and would instead require a re-examination with additional evidence or an independent validation through dating estimates. Second, even though well-established molecular phylogenies have been proposed for praying mantises and termites [17–19], phylogenies including the three groups together had much smaller taxonomic and molecular samples and/or did not incorporate attempts of calibration and datings [16,20]. These limitations impede our understanding of dictyopteran evolution. Yet, the study of this charismatic group of insects, which is deeply rooted in a long chronological timescale [21,22], could shed light on the evolution of a variety of important traits from social or predatory behaviors, to digestive or intracellular symbioses [17,18,23–25]. Our present study aims at understanding the origin of these three groups by overcoming previous limitations in taxon and molecular samplings and in fossil record.

**Fig 1:**
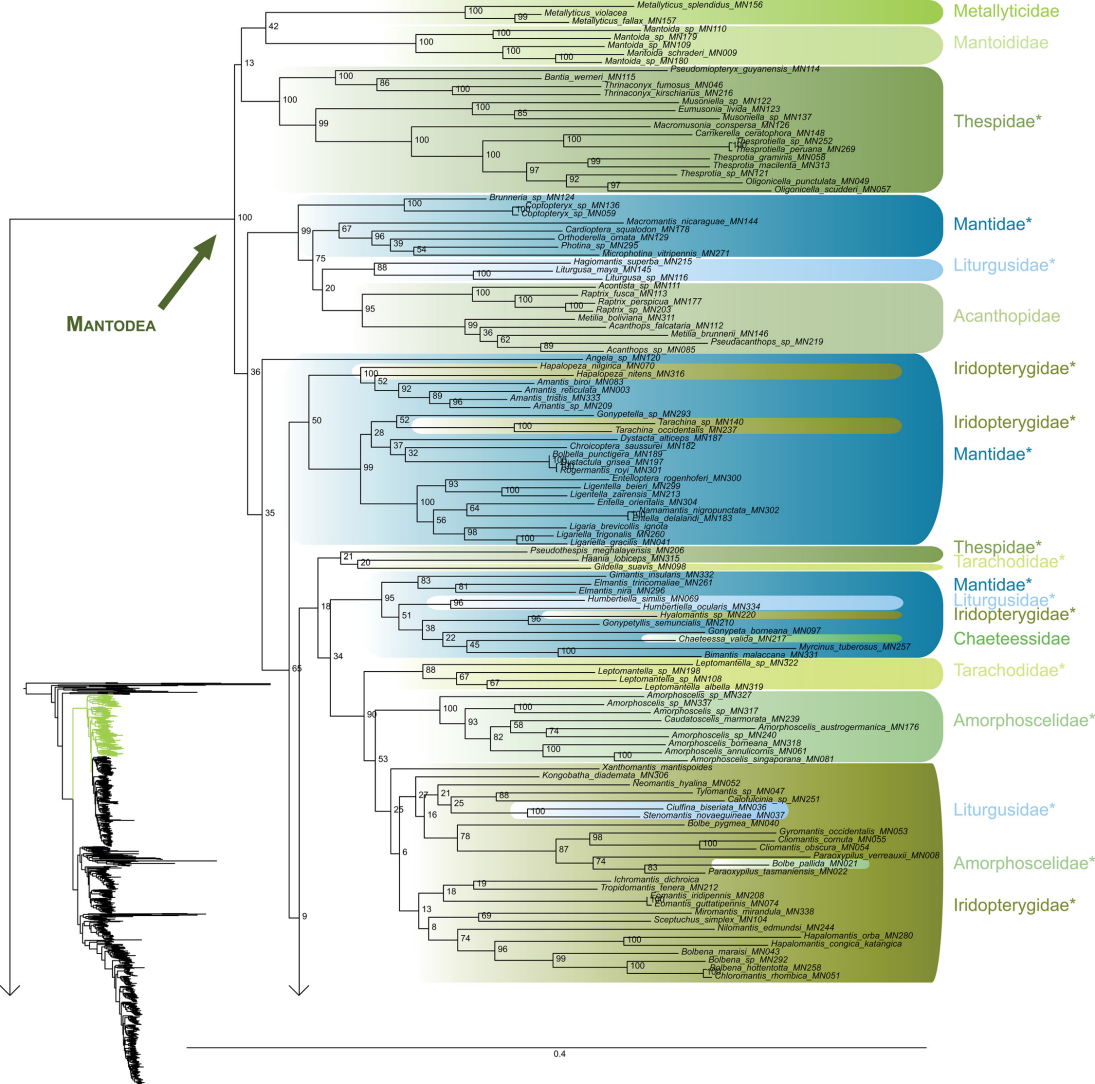
Result of the concatenated analysis of six molecular markers in Maximum Likelihood: mantises. Family names are labeled on the right of the clades. Bootstrap support values are displayed for each node. * = non-monophyletic families.

Several hypotheses exist about the phylogenetic relationships of Dictyoptera or its suborders [16–20,23,25–39]. These works were not all specifically dedicated to test hypotheses of dictyopteran relationships and therefore focused on different taxonomic and character samples. Consequently, directly comparing these phylogenetic hypotheses is intractable but there is one obvious conclusion: we still lack a robust consensus about dictyopteran phylogenetic relationships. For the big picture, the most recent hypotheses converge toward the same general topology for extant species (but see [40,41]): (Mantodea, (other Blattodea, (Cryptocercidae, Isoptera))). However, no study has perfectly replicated previous independent results. In other words, inter-familial relationships are still controversial (see [27]–their ***Fig*** 1). Within cockroaches, authors not only disagree about inter-familial relationships but also about family delimitation. One can potentially postulate up to 11 extant cockroach families but we will follow here Beccaloni and Eggleton [15]. The extant families used are: Blaberidae, Blattidae, Cryptocercidae, Ectobiidae, Lamproblattidae, Nocticolidae, Corydiidae and Tryonicidae. Extinct families also exist and some might rather be stem-Dictyoptera than cockroaches. In termites, Mastotermitidae is undoubtedly sister-group to all other modern termites but disagreements persist over the relationships between Archotermopsidae, Stolotermitidae and Kalotermitidae [17,18,26,42]. As for praying mantises, the most comprehensive study to date [19] has cast serious doubts on traditional taxonomy with nearly half of the accepted families, subfamilies and tribes recovered as non-monophyletic.

Since the nineteenth century, cockroaches are thought to be very ancient because of numerous cockroach-like Palaeozoic and Mesozoic fossils (also called “roachoids”), and traditionally conceived as ancestral to termites and praying mantises [43–48]. The best preserved female “roachoid” fossils show external ovipositors (long or short depending on the taxa), a morphological character that is never found in extant cockroaches. Moreover, other characters such as wing venation (“roachoid” forewings are more frequently preserved than bodies and hindwings) or mouthparts are often incomplete or difficult to interpret. Hence, the systematic relationships of “roachoid” fossils remain disputed. The question, still unresolved, is whether these “roachoids” are indeed true cockroaches or rather a stem-group of Dictyoptera [23,49], even if the Dictyoptera (including these “roachoids”) seems to be monophyletic, with the Palaeozoic Paoliida as its sister group [50]. Placing these fossils requires an adequate outgroup sampling.

Mantises are understood to be much more recent than these “roachoids” according to the fossil record (i.e. Early Jurassic; [25,51,52]). The most recent phylogenetic hypotheses postulate, however, that praying mantises are sister-group to the modern cockroaches or to all other modern Dictyoptera [53]. The relatively young age of the crown group of praying mantises would thus appear contradictory with the hypothesis that all the Palaeozoic and Early Mesozoic “roachoids” could belong to the crown Blattodea. Recently, Béthoux and Wieland [54] and Béthoux et al. [55] found that some Palaeozoic fossils belonging to the family Anthracoptilidae could be stem-mantis lineages, sharing synapomorphic characters with modern praying mantises within the wings and maybe raptorial forelegs. This hypothesis would reconcile the latest molecular phylogenies with the fossil record but it has also been disputed [56–58]. Notably, a recent revision of the Anthracoptilidae [59] suggests that these fossils would belong to the Paoliida, the putative sister-group of Dictyoptera [50].

Finally, termites were always considered as a recent group according to a rich fossil record (i.e. oldest record at the Jurassic/Cretaceous transition; [60–62]). Nevertheless, a few controversial fossil nesting traces would indicate that they are at least 50 My older (i.e. Jurassic or even Late Triassic; [63–67]). Here again, these hypotheses have been criticized [68] and would need proper testing.

Because of these controversies in the fossil record and in phylogenetic hypotheses, these three clades (i.e. cockroaches, praying mantises and termites) all have incongruent dates of origins. Here, we use a supermatrix strategy (about 800 taxa and 10,000 molecular characters) combined with controlled fossil evidence (i.e. considering only fossils attributed to any lineage with clear synapomorphic characters) to overcome the aforementioned limitations. We also nest the strictly dictyopteran ingroup within a comprehensive selection of polyneopteran outgroups for which dating analyses were already published. Thus, we provide a chronological framework of dictyopteran evolution to better estimate the origin and timing of diversification of cockroaches, termites, and praying mantises.

## Materials and Methods

### Taxonomic and character sampling

Given that the most recent molecular phylogenies dealing with all Dictyoptera sub-orders did not sample more than 60 taxa [20,27,39,40], we urge at selecting as many taxa as possible and not subjectively selecting a few of them. Consequently, our taxonomic sample includes 300 praying mantises, 276 termites, 193 cockroaches, and 24 outgroup species, for a total sample size of 793 species. Data are primarily derived from our own works [18,19,25,69,70,71], and Inward’s works on cockroaches and termites [16,17]. We favor here a “supermatrix” rather than a “supertree” approach [72–74]. We supplemented this dataset by generating 210 cockroach sequences to improve their representativeness and with data available on GenBank, providing that at least three markers were included for each species in the analysis, to limit potential reconstruction artifacts due to missing data. For the generated sequences, we notably focused on families and subfamilies that were previously poorly sampled including: Blattidae (25 species), Pseudophyllodromiinae (10 species), Corydiidae (four species), Nocticolidae (one species), Anaplectinae (one species) and Lamproblattidae (one species). All newly generated sequences were submitted to GenBank and their accession numbers (KP986236-KP986445) are provided in S1 Table. Molecular protocols are detailed in Legendre et al. [18].

For character sampling, we selected molecular markers that were documented for at least 50% of the taxa, which includes four mitochondrial [12S rRNA (~ 380 bp), 16S rRNA (~ 480 bp), and cytochrome oxidase subunits I (~ 1280 bp) and II (~ 650 bp)–hereafter COI and COII] and two nuclear markers [18S rRNA (~ 1800 bp) and 28S rRNA (~ 2000 bp)]. The full data set includes 3674 sequence fragments from these six loci. We sampled 92.7% of the taxa for the 12S, 66.8% for the 16S, 58.6% for the COI, 89.9% for the COII, 69.4% for the 18S and 85.9% for the 28S. We sampled 41.1% of the taxa for the six markers, 11.5% for five markers, 17% for four markers and 30.4% for three markers. The intensity of data completeness within each suborder differs: 96% of praying mantises are documented for at least five markers, whereas it concerns 35% and 17% of cockroaches and termites, respectively. Details are provided in S1 Table.

For outgroup comparison we used modern taxa belonging to the different polyneopteran clades (Dermaptera, Embioptera, Grylloblattodea, Mantophasmatodea, Orthoptera, Phasmatodea, and Plecoptera) and Ephemeroptera as rooting outgroups [53]. Zoraptera affinities are not clear [53] and recent works postulate that they could be the sister-lineage of Dictyoptera [75,76]. Thus, we initially included a Zoraptera species (Zorotypus novobrittanicus) within outgroups. It was, however, removed from final analysis because, in preliminary analyses, Z. novobrittanicus was included within Blattodea as the sister lineage of Xestoblatta sp.1, which was undoubtedly artifactual (likely due to contamination issues in molecular sequences; S. Cameron, pers. comm.). We therefore removed Z. novobrittanicus from the dataset based on its behavior as a wildcard taxon in our analyses.

### Alignments and phylogenetic analyses

We used the software MUSCLE 3.8 [77] to align molecular sequences. Because the sequences used here come from different studies and were thus generated with an assortment of primers, the fragments were not always congruent in coverage, which generated a few dubious alignments for some sequences in the terminal regions. We corrected these problems by refining the alignment manually. We also checked that alignments for protein-coding genes were congruent with codon reading frame using BioEdit 7.0.5.3 [78]. As described in Legendre et al. [18], 28S rRNA was partitioned into four sequences to optimize automatic alignment with MUSCLE and to limit eye-driven homology hypotheses correction. We used the software SequenceMatrix 1.7.7 [79] to concatenate the supermatrix. It resulted in a final alignment of approximately 10 kbp (S1 Dataset).

Maximum likelihood analyses were conducted using RAxML 7.2.8 [80] with a GTR + Γ model. We did not consider models mixing a proportion of invariant sites (I) with a gamma distribution shape parameter (Γ) because these two parameters are strongly correlated [81], which could bias the estimation of these parameters. We used Mrmodeltest 2.3 [82] under the Akaike Information Criterion [83,84], which selected the GTR + Γ model as the most appropriate model that does not combine I and Γ. We first run separate analyses to check for obvious artifacts or contaminations. We then performed 100 ML replicates using the rapid hill-climbing algorithm on the combined dataset and the optimal solution was selected. Suboptimal solutions were kept to run dating analyses on and to obtain confidence intervals (see below). We estimated support values based on 100 bootstrap replicates using the rapid bootstrap algorithm [85] implemented in RAxML. All analyses were performed on a HP Z800 Workstation with 17.9 GB RAM and an Intel Xeon CPU E5520, using six or seven threads.

Bayesian Inference via MrBayes was performed on both the cluster of the Paris Museum and the Cleveland Museum of Natural History analytical server, but both were hampered by memory limitations and time to complete the analyses. It was thus impossible to include such analyses in spite of calculation attempts of several months.

### Fossil calibrations and molecular datings

Uncertainties about the timing of diversification in Dictyoptera are due in part to incomplete or controversial fossils. We did not use these controversial fossils but tested them with a conservative approach, wherein the possibility to infer old age estimates was kept as follows.

First, we placed a maximal age constraint at the root, which corresponds to the differentiation between Palaeoptera and Neoptera, of 470 Mya. This value corresponds to the maximum of the 95% confidence interval inferred in Rehm et al. [86] in their dating of the Arthropod tree. This is a very old limit given that the Palaeoptera/Neoptera diversification is commonly thought to have occurred around 400 Mya [87]. Using a maximal age constraint at the root is a common strategy used to avoid artifactual old age estimates of the root with the PL method [88].

Second, we used 17 fossils as minimum age constraints as calibration points (Table 1). We chose these fossils because we considered that we were able, according to their descriptions, to reliably assign them to a node in our recovered phylogeny. Fossils that could not be assigned unambiguously to a particular lineage (e.g., no accurate synapomorphy of the concerned clade in the description of the fossil) were not included [8]. In particular, fossils from extinct “roachoid” families (e.g., Mesoblattinidae, Phylloblattidae) are among those not included in our calibration points. One fossil (Arverineura insignis) has a peculiar situation because both its placement as stem Chaeteessidae and the position of Chaeteessa valida (single Chaeteessidae sampled here) in the phylogeny (see below) can be criticized. Arverineura insignis is only known by a forewing and its venation is nearly identical to that of Chaeteessa valida so that Nel and Roy [89] suggested they could be the same genus. The presence of an oblique pseudo-vein (stigma of [90]) in the mid part of the forewing or the fact that the most posterior branch of CuA is simple could be apomorphies, but the polarization of these character states remains an issue. We thus also ran additional dating estimates without Arverineura insignis to check if its inclusion in the analyses had an impact or not.

**Table 1:** Details about the fossils used as calibrations (minimal ages) in the dating analyses.

Species	Age (Ma)	Phylogenetic position	Reference	Museum specimen number	Apomorphy	Locality and stratigraphy	Reference to a published age
Gulou carpenteri	315	stem Plecoptera	[134]	CNU-NX1-143	Presence of a broad MP/CuA and CuA/CuP areas in forewings, with a series of parallel simple crossveins	Qilianshan entomofauna, locality of Xiaheyan Village (Zhongwei City, Ningxia Hui Autonomous Region, China); Tupo Formation, Pennsylvanian strata, Bashkirian	[135]
Qilianiblatta namurensis	315	stem Dictyoptera	[124]	GMCB 04GNX1001-1	Presence of a deeply concave CuP in forewing [50]	Qilianshan entomofauna, locality of Xiaheyan Village (Zhongwei City, Ningxia Hui Autonomous Region, China); Tupo Formation, Pennsylvanian strata, Bashkirian	[135]
Juramantophasma sinica	158	stem Mantophasmatodea	[118]	NIGP 142171 (Nanjing Institute of Geology and Palaeontology, Chinese Academy of Sciences)	A third tarsomere with a sclerotized elongated dorsal process; enlarged and fan-like pretarsal arolia with a clearly visible row of dorsal setae; last tarsomere making a right angle with the others, keeping it up in the air; female gonoplacs (valves 3) short and claw-shaped; and egg with a circular ridge	Daohugou, Ningcheng County, Inner Mongolia, NortheastChina; Jiulongshan Formation, Middle Jurassic (Callovian/Oxfordian)	[136]
Mastotermes nepropadyom	140	stem Mastotermitidae	[62]	PIN 4626/156 (Moscow)	Hindwing with Mastotermes-like anal field	Chernovskie Kopi, Chita Region, Chita District, left bank (stream side) of the Ingoda River; Doronino Formation, Chernovskaya transitional sequence; Upper Jurassic–Lower Cretaceous.	[137]
Piniblattella vitimica	130	stem Ectobiidae	[122]	PIN 1989/1639, 1646 (Moscow)	Fanlike fold in hind wings (when present) does not include the first four rami; conspicuous tergal glands (not in all but only in some Ectobiidae)	Baissa (Russia), Zaza formation; Lower Cretaceous, supposedly earliest Berriasian-Valanginian	[138]
Cretaholocompsa montsecana	125	stem Holocompsinae	[121]	LC-1704-IEI	No vein in medio-distal part of forewings	La Cabrua outcrop, Sierra del Montsec (Spain); Pedrera de Rubies Formation, Barremian	[139]
Cratokalotermes santanensis	112	stem Kalotermitidae	[140]	SMNS 66195	Crowded radial field and long cubital field (extends to near the apex of the wing)	Crato, Santana Formation (Brazil), Early Cretaceous (Aptian)	[139]
Morphna paleo	62	stem asian Epilamprinae	[141]	PIN 5142/12	The combination of parallel forewing margins, wide and branched Sc, fusion of M with CuA running close to R, basalmost branches of CuA running parallel to CuP and simple A	Archara-Boguchan, Far East, Russia; Tsagayan Formation, Danian Paleocene	[142]
Arverineura insignis	60	stem Chaeteessidae	[89]	MNHN-LP-R.07020 (specimen 715, Piton coll.)	Presence of an oblique pseudo-vein in the mid part of the forewing? most posterior branch of CuA simple? *	Menat (France), Menat Formation (Piton collection), Thanetian	[143]
Prochaeradodis enigmaticus	60	stem Choeradodinae	[89]	MNHN-LP-R-07003	Broad side lobes of the pronotum and reticulated forewing with a very wide costal area.	Menat (France), Menat Formation (Piton collection), Thanetian	[143]
Nanotermes isaacae	50	stem Termitidae	[144]	BSIPL Tad-262 (Lucknow, India)	Radial vein simple + reduction of M + CuA with a series of simple posterior branches	Tadkeshwar lignite mine (India: Gujarat); Cambay Formation, Ypresian	[145]
Archotermopsis tornquisti	41	crown Archotermopsidae	[60,61,126,146]	1133, Typ. Kat. Nr. 255	Absence of ocelloids and fontanelle, antennae with 22–27 articles, pronotum distinctly narrower than head, tarsi pentamerous (sometimes cryptically), fourth sternite with sole sternal gland, forewing scale overlapping hind-wing scale, humeral margin of scale flat, imago-worker mandibles with three marginal teeth (left side) and subsidiary tooth between apical and first marginal teeth (right side)	Kaliningrad (Russian Federation); Baltic amber, middle Eocene (Lutetian)	[147]
Heterotermes eocenicus	41	stem Heterotermes	[148]	B-163	Wing membrane setae present, microsetulose + Imago compound eye small, not protruding beyond lateral margin of head in frontal view + Imago ocelloid small, ca. 2–3x diameter of compound eye facet	Kaliningrad (Russian Federation); Baltic amber, middle Eocene (Lutetian)	[147]
Ulmeriella rubiensis	28	stem Hodotermitidae	[149]	B-72	One (or two) posterior branch(es) subapical of R and well-developped	Ruby River Site 1, Montana (USA); Passamari Formation, Rupelian	[150]
Dolichorhinotermes apopnus	20	stem Dolichorhinotermes	[151]	AMNH Ch-50—Amber Fossil Collection, Division of Invertebrate Zoology, American Museum of Natural History	Imago with third flagellar article shorter than first flagellar article. Major soldier with labrum distinctly elongate, apex of labrum frequently extending to mandibular apex. Minor soldier with opening of frontal gland at front of head but not on conspicuous prolongation of head capsule; mandibles vestigial, with rounded margins; sides of head in dorsal aspect straight or convex	Simojovel (Mexico: Chiapas), Chiapas amber; Early Miocene	[152]
Holocompsa nigra and H. abbreviata	15	stem Holocompsa	[153]	NMNH, no. 502411, Acc. 371428, Woodruff (collection reg.) 3751, Brodzinsky / Lopes-Pena Collection (H. nigra) / NMNH, no. 504367, Acc. 371428, Woodruff (collection reg.) 8813, Brodzinsky / LopesPena [Penha] Collection	Head with a two-parts very large clypeus reaching the antennal sockets, small body size, hind wings with specialized venation	Dominican amber (USNM Brodzinsky Lopez-Pena coll); Miocene, Burdigalian/Langhian	[154]
Constrictotermes electroconstrictus	15	stem Constrictotermes	[155]	AMNH DR-14-584	Head constricted, characteristic of Constrictotermes	Dominican Republic amber, specific locality not known; Miocene, Burdigalian/Langhian	[154]

* the oblique pseudo-vein in the mid part of the forewing is reduced in the Mantoididae and of different shape in Metallyticus. The most posterior branch of CuA is not simple in the Mantoididae and of different shape in Metallyticus [90,156].

Molecular dating analyses were computed with r8s 1.71 [91]. As for phylogenetic reconstructions, it was impossible to compute divergence estimates in a bayesian framework due to computational limitations, a problem faced in other studies with large taxon sample (e.g., [92,93]). In addition, multiple empirical studies at different scales (e.g., [94–96]) suggest that r8s estimates usually strongly overlap with BEAST estimates [97], especially with low values of smoothing, for which much rate variation is permitted (i.e. non-clocklike data). Finally, autocorrelated models proved to have a higher statistical fit to the data than uncorrelated models [98,99].

We used ML trees with the penalized likelihood (PL) method [100] using the TN algorithm and a logarithmic penalty function. A cross validation procedure was performed to choose the optimal value of smoothing. Nevertheless, after more than a month of analysis, only three smoothing parameter values have been tested during this procedure. Therefore, we decided to follow two alternative, quicker, strategies. First, we ran two cross validation procedures with an additive penalty function (four smoothing values, λ, between 1 and 1000, and five smoothing values between 1 and 2.5 – values of λ < 1 were also tested but the analyses failed, which is a known possible issue of the algorithm with extremely low smoothing parameters [100]). In both cases, a rate smoothing parameter of 1 had the lowest chi-square value. Second, with a logarithmic penalty function, we performed ten dating analyses with different smoothing parameter values (1, 10, 35, 50, 80, 125, 200, 400, 900 and 2000) to check how this parameter impacts the dating estimates, especially for the deepest nodes as they are the most important for our study. We selected this intermediate range of smoothing values because it is generally with these values that cross validation scores are most optimal [88,100]. The biggest date estimate difference uncovered between different smoothing values was of 22 My for the deepest nodes, with older age estimates corresponding to lower smoothing values (S2 Table). This difference was considered as relatively low. Then, to perform the subsequent dating analyses, we chose a smoothing value of 1. This choice results from the cross validations and fits our conservative approach of hypotheses testing because smaller values of smoothing resulted in older age estimates for our data.

Note that the PL method cannot deal with very short branches (smaller than 0.0000099 for our trees; [101]). Consequently, for each tree used in dating analysis, and following the recommendation found in r8s manual, we removed these very short branches using the R package ape (command ‘drop.tip’; [102]). Taxa supported by these very short branches have been identified and reported in a tab delimited file, which was imported into R. This file and the script used afterwards are provided in Supporting Information (S1 and S2 Methods). These few branches (mean +/- SD = 9 +/- 2) were mainly terminal branches leading to species representing genera with multiple representatives, so their exclusion did not impact on generic sampling and dating estimates.

Finally, we estimated approximated 90% confidence intervals by repeating the dating procedure 100 times with 100 trees coming from our 100 ML analyses (see Alignments and phylogenetic analyses). We thus took into account potential sources of error in dating estimates due to phylogenetic uncertainty (both in tree topology and branch lengths). These confidence intervals were calculated using the R package Locfit [103] and following the procedure detailed in Lopez-Vaamonde et al. [104].

## Results

### Phylogenetic analyses

**Fig 2:**
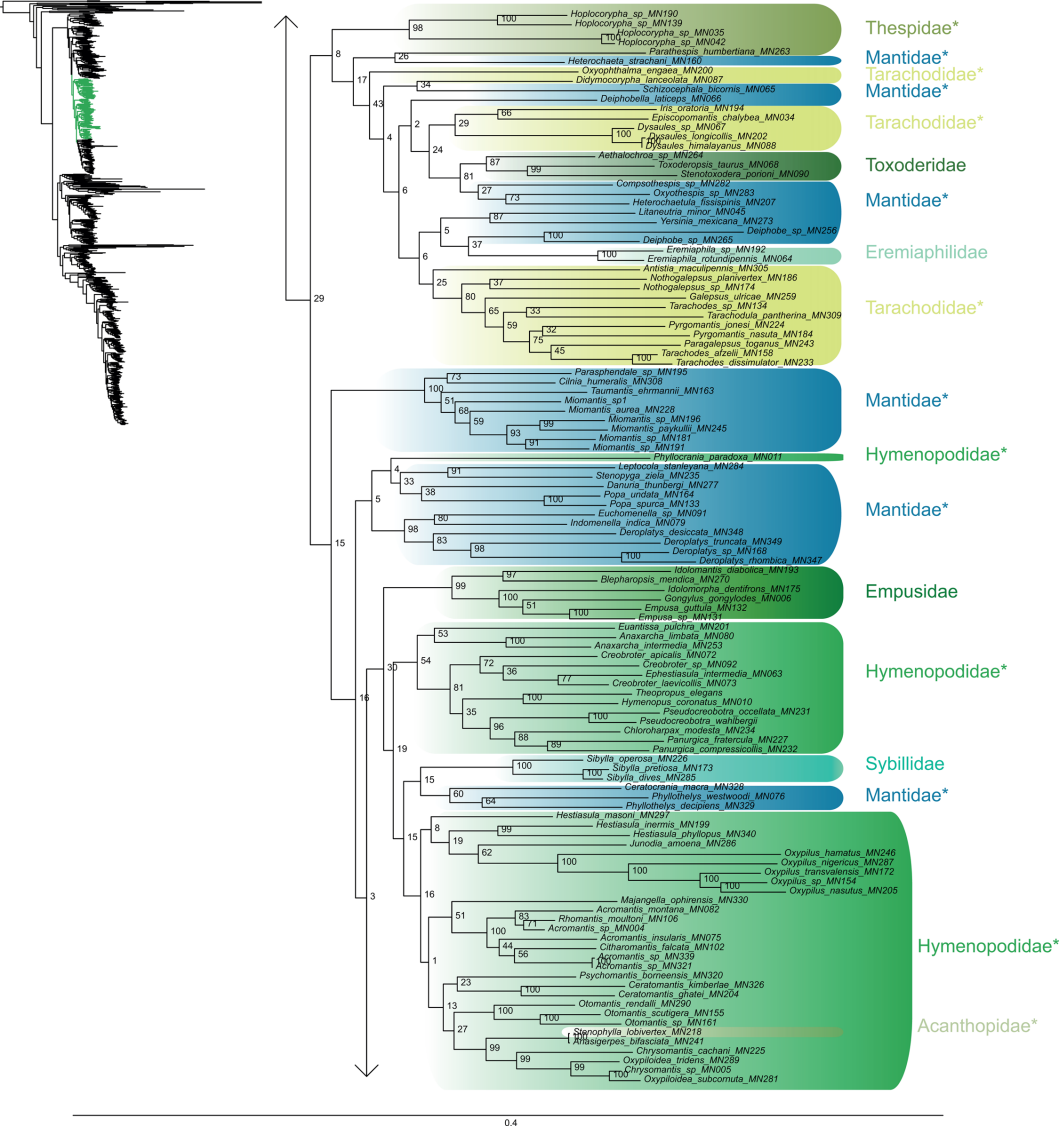
Result of the concatenated analysis of six molecular markers in Maximum Likelihood: mantises (continued). Legend as in Fig 1.

**Fig 3:**
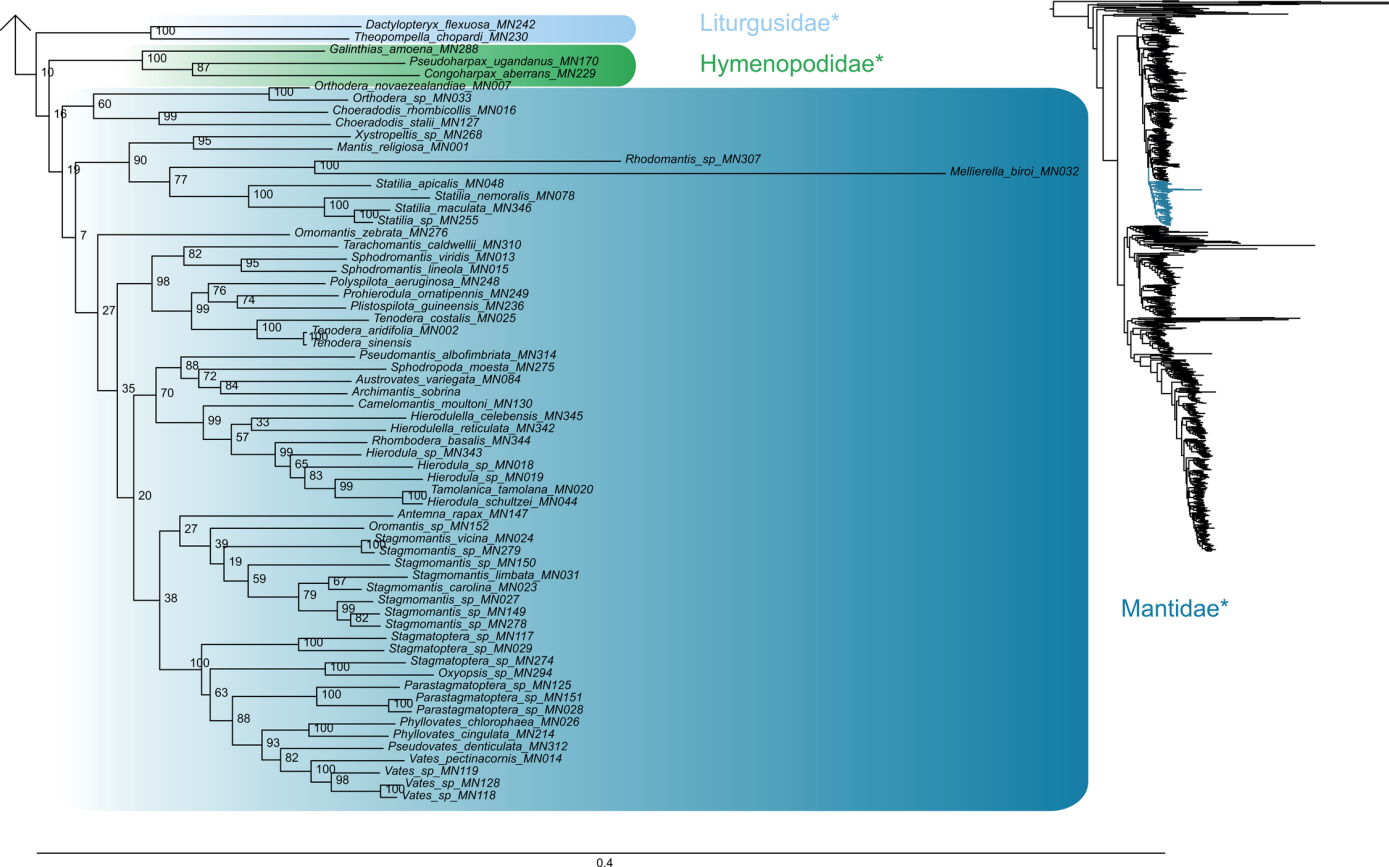
Result of the concatenated analysis of six molecular markers in Maximum Likelihood: mantises (continued). Legend as in Fig 1.

**Fig 4:**
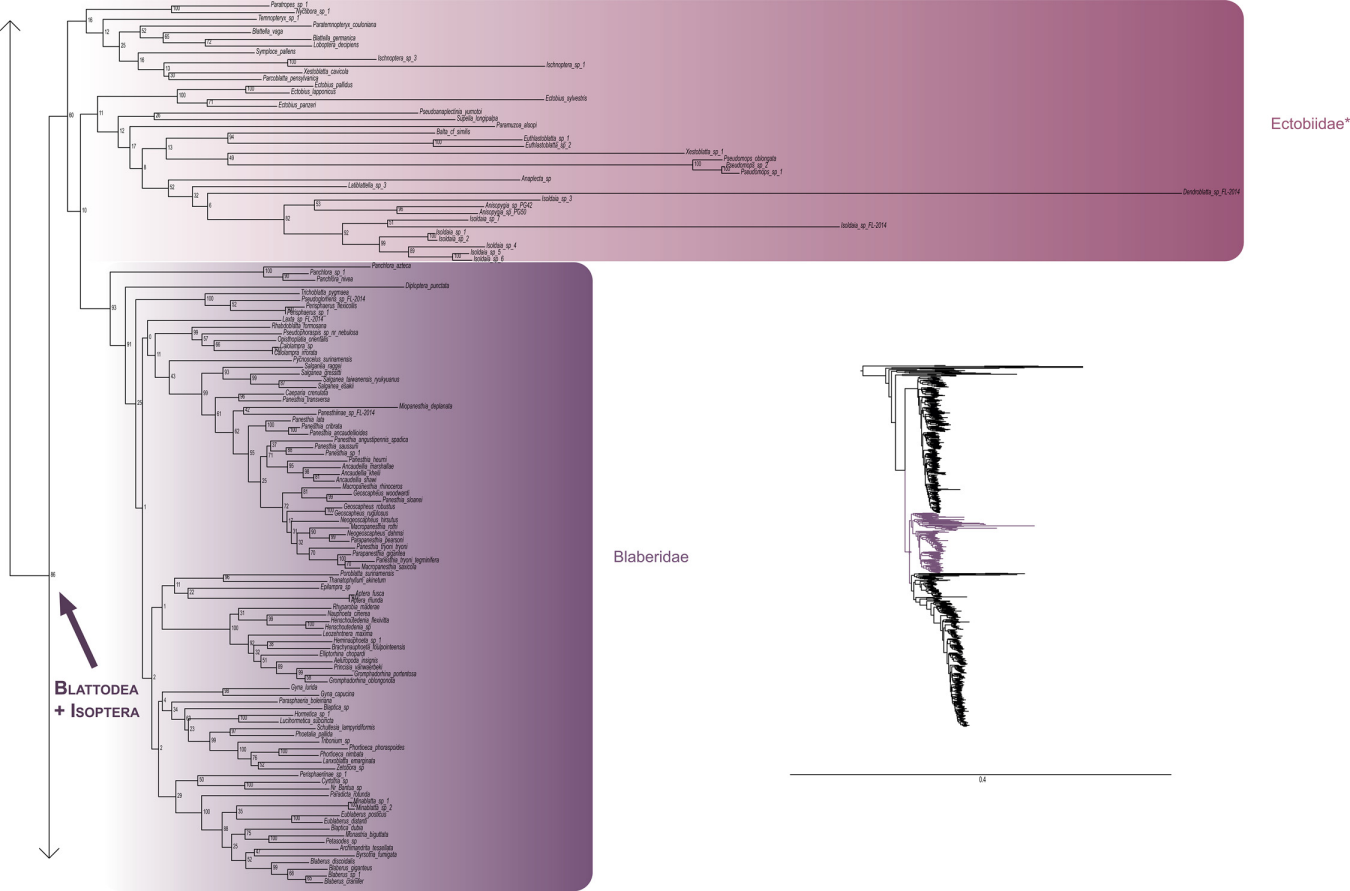
Result of the concatenated analysis of six molecular markers in Maximum Likelihood: cockroaches. Legend as in Fig 1.

**Fig 5:**
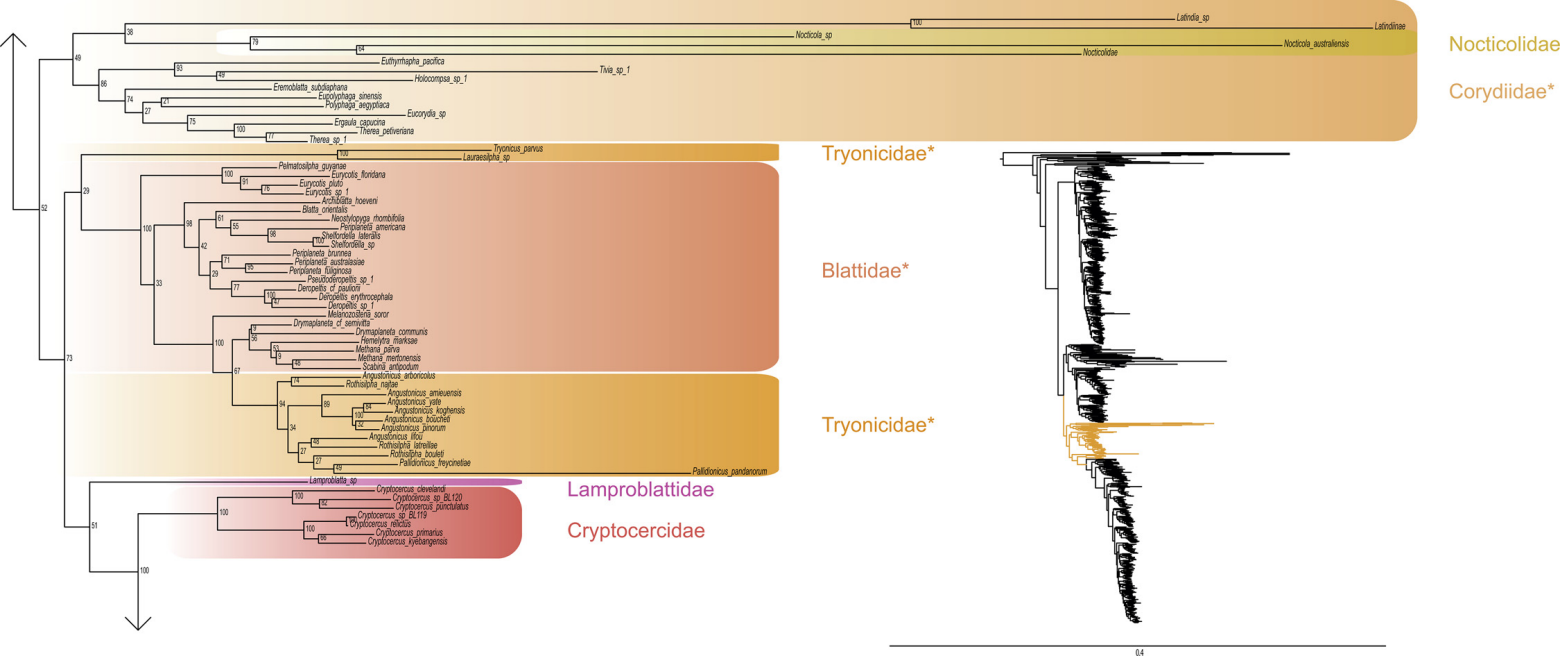
Result of the concatenated analysis of six molecular markers in Maximum Likelihood: cockroaches (continued). Legend as in Fig 1.

**Fig 6:**
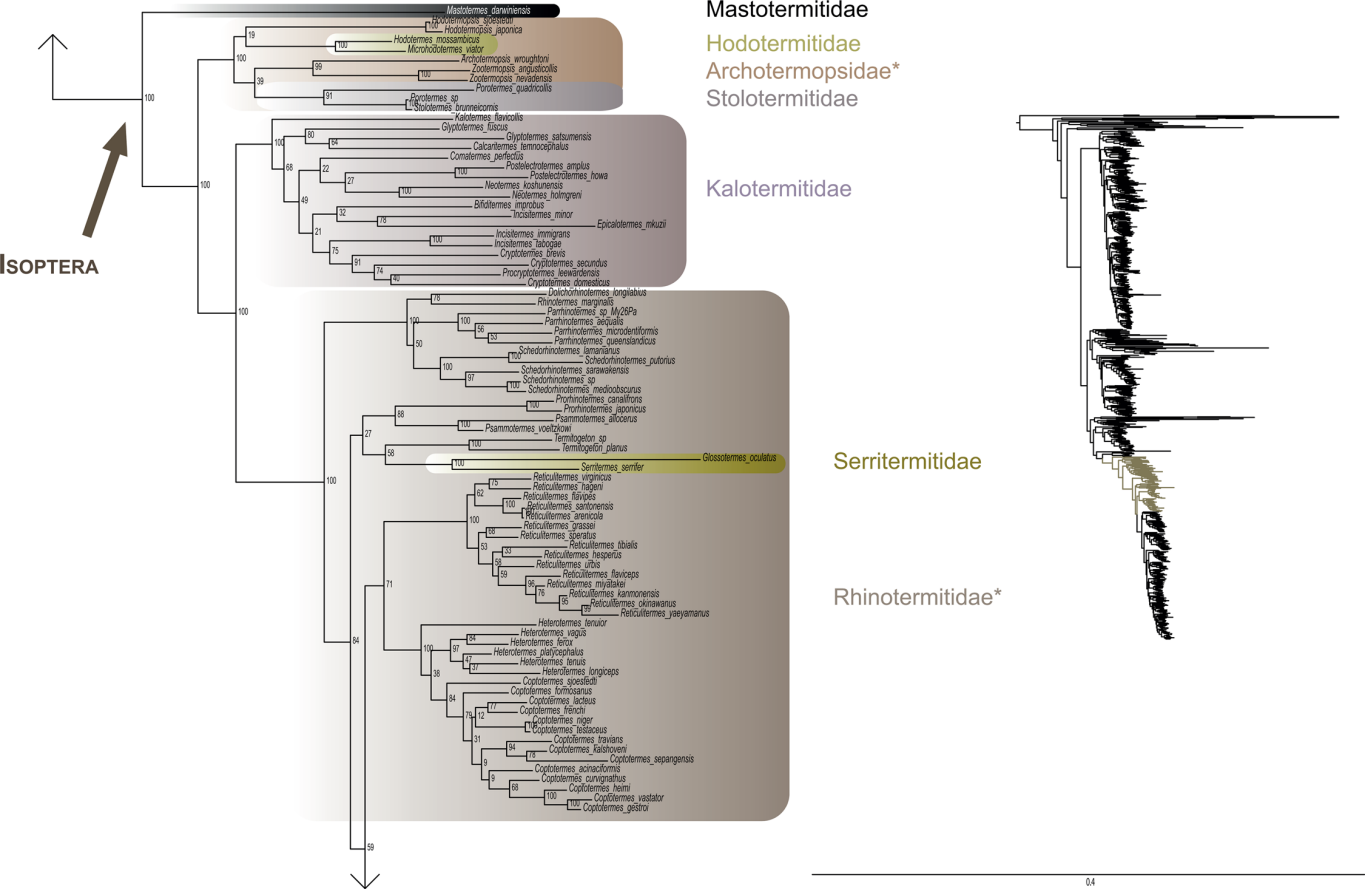
Result of the concatenated analysis of six molecular markers in Maximum Likelihood: termites. Legend as in Fig 1.

**Fig 7:**
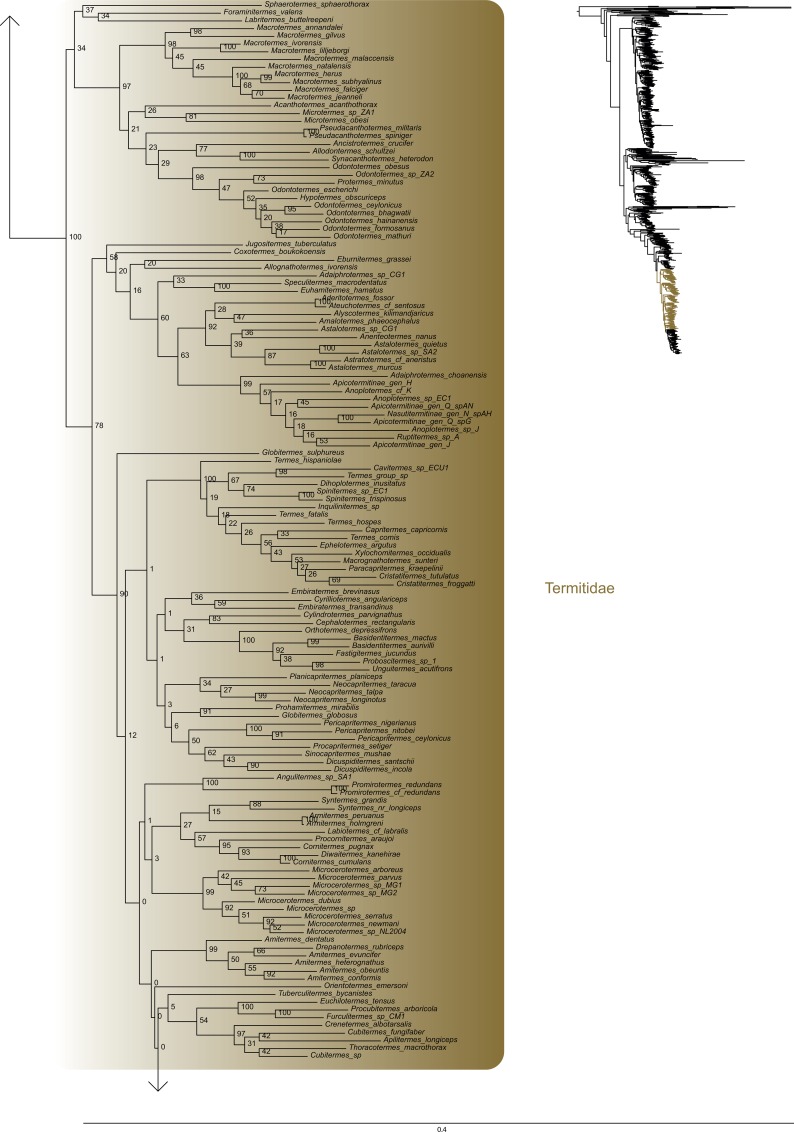
Result of the concatenated analysis of six molecular markers in Maximum Likelihood: termites (continued). Legend as in Fig 1.

**Fig 8:**
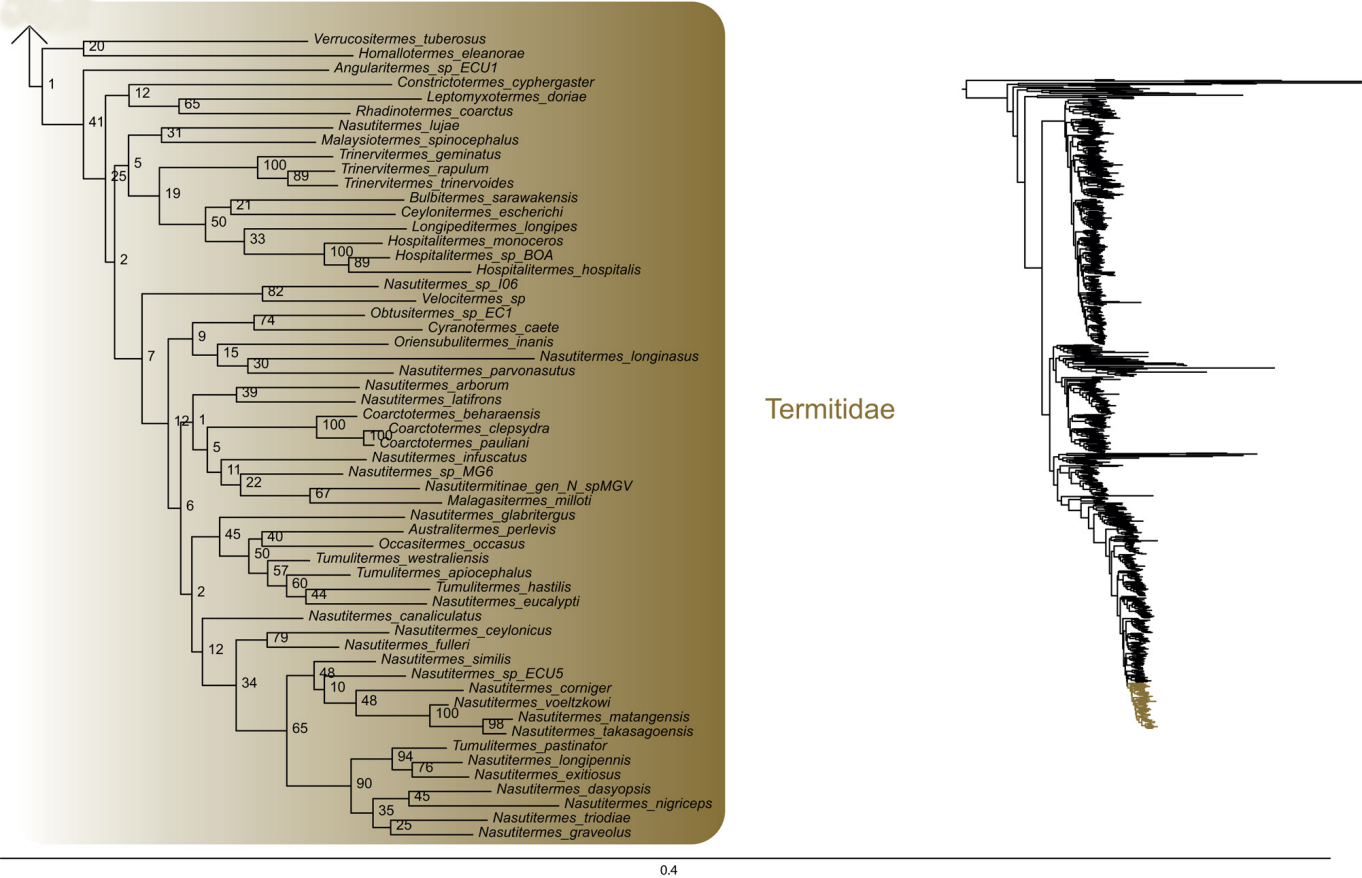
Result of the concatenated analysis of six molecular markers in Maximum Likelihood: termites (continued). Legend as in Fig 1.

The most likely tree (Figs 1–8; ln L = -627407.49) recovered Dictyoptera, Mantodea and Isoptera as monophyletic groups with maximal support values (i.e. bootstrap support of 100), whereas Blattodea was paraphyletic. The group (Blattodea + Isoptera) was monophyletic with a high support value (BS = 86). Our results were congruent with the most recent hypotheses about inter-order relationships. Even though our aim was not to propose a classification, we found some original intra-ordinal relationships detailed hereafter.

The early branching order of Mantodea (Figs 1–3) included a monophyletic Mantoididae with Metallyticidae (BS < 50) while Chaeteessidae was deeply nested within Amelinae. Most of the families were recovered as paraphyletic including Hymenopodidae, Mantidae, Thespidae, Iridopterygidae, Liturgusidae, Amorphoscelidae, and Tarachodidae. Few families were monophyletic, which include Acanthopidae (BS = 95), Empusidae (BS = 99), Eremiaphilidae (BS = 100), Mantoididae (BS = 100), Metallyticidae (BS = 100) and Toxoderidae (BS = 87).

Blattodea (Figs 4 and 5) split up into two groups. One group was monophyletic (BS = 60) and included Ectobiidae and Blaberidae. The other group was paraphyletic and included Corydiidae, Nocticolidae, Blattidae, Tryonicidae, Lamproblattidae, and Cryptocercidae. In the first group, Anaplectinae–represented only by Anaplecta sp.–was nested within Ectobiidae, as sister taxon of several Pseudophyllodromiinae. Blaberidae was monophyletic with a high support value (BS = 93), whereas Ectobiidae was paraphyletic. In the second group, the monophyletic Nocticolidae was closely related to Latindiinae within Corydiidae, which is paraphyletic. Lamproblatta sp. was the sister taxa of (Cryptocercus spp. + Isoptera) but this relationship was not well-supported (BS = 51). The clade (Tryonicus parvus + Lauraesilpha sp.) was sister taxa to a clade comprising the Blattidae and the remaining Tryonicidae, a result weakly supported (BS < 50). The monophyly of (Blattidae + Tryonicidae) excluding Tryonicus parvus and Lauraesilpha sp. was highly supported (BS = 100), as was the sister-group relationship of Cryptocercidae with Isoptera (BS = 100).

Within termites (Figs 6–8), Mastotermitidae was sister-group to all other modern termites with maximal support value. Then, the clade (Hodotermitidae + Archotermopsidae + Stolotermitidae), which was highly supported (BS = 100), was sister-group to all the remaining termites. Kalotermitidae was monophyletic (BS = 100) and was sister-group to (Rhinotermitidae + Termitidae), a result highly supported (BS = 100). Serritermitidae (here Serritermes serrifer and Glossotermes oculatus) was monophyletic (BS = 100) and was nested within paraphyletic Rhinotermitidae.

Finally, from the global picture, we noted that internal branches within Blattodea were, with our data set, longer than those within Mantodea and Isoptera. In particular, there were two remarkable clades with long branches: one within Ectobiidae (Fig 4) and the other within (Corydiidae + Nocticolidae) (Fig 5). The first one mainly dealt with Pseudophyllodromiinae; the second one dealt with Latindiinae and Nocticolidae species.

### Dating analyses

**Fig 9:**
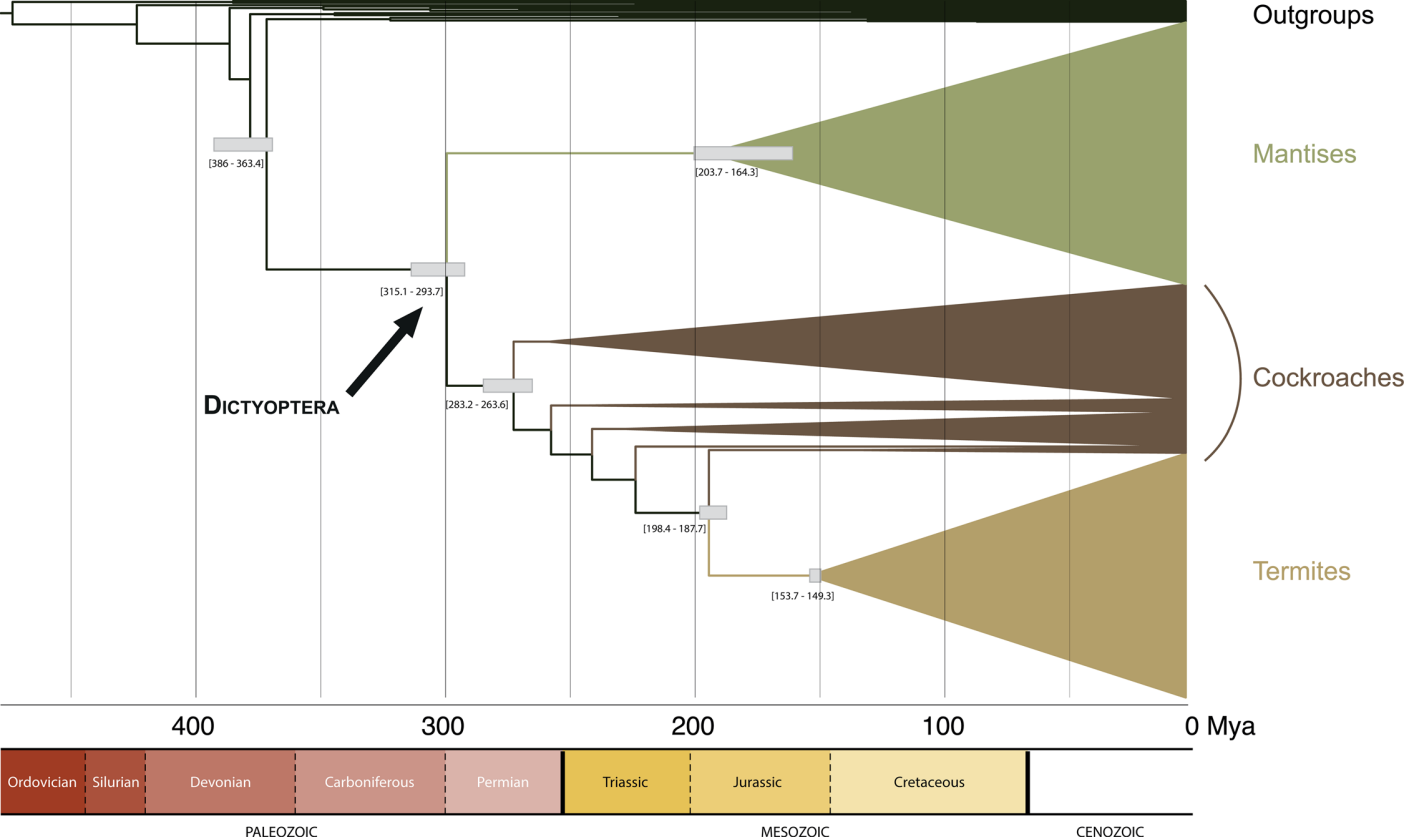
Simplified chronogram obtained in Penalized Likelihood dating analyses. Grey bars represent approximated 90% confidence intervals.

Our dating estimates are provided as a simplified chronogram in Fig 9. They suggested that stem-Dictyoptera would date back to the Middle-Late Devonian (mode = 382 Mya range = 363–386.4 Mya). Crown-Dictyoptera would have originated around the Carboniferous/Permian boundary (mode = 300.7 Mya; range = 293.7–315.1 Mya). Crown-group diversification of praying mantises would have occurred in the Early-Middle Jurassic (mode = 191.8 Mya; range = 164.3–203.7 Mya). Stem-termites would date back to the Early Jurassic (mode = 192.2 Mya; range = 187.7–198.4 Mya). Crown termites diversification would date back to the Late Jurassic (mode = 151.3 Mya; range = 149.3–153.7 Mya). Finally, the most recent common ancestor of (Blattodea + Isoptera) would date back to the Permian (mode = 270.9 Mya; range = 263.6–283.2 Mya). Results without the fossil calibration based on Arverineura insignis (stem Chaeteessidae node) were very similar (dating estimates difference of 2.3 Mya at most).

## Discussion

### Dictyopteran phylogenetic relationships

Dictyoptera is monophyletic, which is a hypothesis supported by multiple morphological and molecular studies (e.g., [32,49,105–108]). Within Dictyoptera, intra-ordinal relationships are congruent with the most recent molecular phylogenetic studies [16,20,39] with monophyletic praying mantises and termites, and paraphyletic cockroaches. For these three groups, we face different situations from a taxonomic and character samplings point of view when compared with previous molecular studies: our praying mantis data set is mainly a subsample of Svenson and Whiting [19]; our termite data set is mainly a combination of Inward et al. [17] and Legendre et al. [18]; our cockroach data set brings several new taxa. Consequently, our phylogenetic results are more worth discussing for cockroaches and termites than for praying mantises.

The recovery of paraphyly in many of the higher-level groups of Mantodea is not surprising and congruent with previous studies [19,25]. The major clades, which mostly include paraphyletic groups of families, subfamilies and tribes, are not significantly different from those found by Svenson and Whiting [19], and the composition of the clades is largely the same. However, the branching of these major clades as well as the early branching order of Chaeteessidae, Mantoididae, and Metallyticidae are different in our results. The recovery of Metallyticidae with Mantoididae is unique to our study and may have resulted from the influences of outgroup sampling and the dubious placement of Chaeteessidae. The latter issue was also found in previous analyses [71] and it seems to resolve better with more data [19]. Regardless of the various topological differences between our phylogeny and the one published by Svenson and Whiting [19], the classification is at odds with the molecular and morphological phylogenies (see [90]).

Within cockroaches, we find two main groups. In the Blaberoidea clade, the Blaberidae family is monophyletic and sister-group of an assemblage of some Ectobiidae subfamilies, the Ectobiidae being paraphyletic. This pattern, apart from the phylogenetic position of Anaplectinae that has rarely been investigated, was repeatedly suggested in the recent molecular literature [16,20,27,39,41] even though the topologies found all differ and are never consistent with the patterns of Ectobiidae paraphyly as previously proposed on a morphological basis [23,30–32,36]. Anaplectinae is here nested within Ectobiidae, whereas it was placed as sister-group of all other Blaberoidea (i.e. Blaberidae + Ectobiidae) on a morphological basis [23,32,36], or with a close affinity to (Cryptocercidae + Isoptera) or Tryonicidae [20]. A larger sampling of this worldwide subfamily is required to assess and discuss further its phylogenetic position. In the second clade, Nocticolidae and Latindiinae are monophyletic, whereas Corydiidae is paraphyletic. A close relationship between these three lineages was suggested by Grandcolas [23] on the basis of morphological characters, and also found more recently with molecular and morphological data [20]. Tryonicidae are not monophyletic, as in Murienne [37] but contrary to Grandcolas [109]. Nevertheless, the sister-group relationship between Tryonicus and Lauraesilpha is congruent with Grandcolas [109], Murienne et al. [110] and Murienne [37]. The Blattidae and the remaining Tryonicidae form a well supported group with a phylogenetic position compatible with the one found in Inward et al. [16], but not with the phylogenetic hypothesis of Murienne et al. [110] or Djernaes et al. [20,27]. The phylogenetic position of Lamproblatta as sister-group to (Cryptocercus + Isoptera) is an original, unexpected and poorly supported result, which would deserve further investigation as Lamproblattidae species are too rarely included in phylogenetic analyses, especially with molecular data (but see [20,23,27,32]). Cryptocercidae is monophyletic and sister-group of Isoptera, a result congruent with most recent molecular analyses (e.g., [16,18,26,42,107]) but contrasting with Gäde et al. [111] and Grandcolas [23,112].

Within termites, our analyses suggest that (Archotermopsidae + Stolotermitidae + Hodotermitidae) is sister-group to all termites but Mastotermitidae ([17,26,42] but contra [18]). It is the first time that such a relationship is strongly supported with both molecular data and more than four species. Serritermitidae is nested within Rhinotermitidae as sister-group of Termitogeton, a result already found in Inward et al. [17], Legendre et al. [113] and Bourguignon et al. [26].

Two clades within Dictyoptera are particularly worth noticing given their very long branches: Ectobiidae and (Corydiidae + Nocticolidae). These long branches suggest dramatic evolution rate changes within these cockroach clades for some or all of the molecular markers used. This phenomenon should be investigated further and be considered in future phylogenetic studies of cockroaches as it might affect tree reconstruction [114–116].

### Timing of diversification in Dictyoptera and fossil data

We used a conservative approach allowing old age estimates with double-checked fossils and a large phylogenetic sample to illuminate the timing of diversification of Dictyoptera and of its three sub-orders (i.e. Blattodea, Mantodea and Isoptera). This approach aimed at testing whether the presumptive old ages of each sub-order could be confirmed or refuted. We tested (1) the origin and diversification of praying mantises, with regard to recent but controversial fossil data that pushed back the origin of this group for more than 150 My [54]; (2) the origin of Blattodea, which provides a chronological framework to fit Palaeozoic and Mesozoic “roachoids” in; (3) the origin of termites, which brings substantial information about eusociality evolution.

### Timing of diversification in praying mantises and palaeoecological implications

Whereas the origins of praying mantises have been thought to be rather recent (~ 150 Mya–Grimaldi, 2003), it has been dramatically pushed back in time following recent fossil discoveries. The Late Carboniferous-Early Permian origin of crown-Dictyoptera inferred here is compatible with the Carboniferous Anthracoptilidae, the putative stem-mantodeans (~310 Mya; [54,55]). However, this compatibility does not resolve the debate concerning the interpretation of these fossils (e.g., [56–59]), in which some authors suggest that they rather belong to Hypoperlida or Eoblattidae. Moreover, our recovered estimates are significantly older than those recovered by Svenson and Whiting [19] and Misof et al. [117], where stem-mantises dated to the Triassic-Jurassic boundary (~ 200 Mya) and crown diversification occurred in the Late Jurassic (~150 Mya; [19]) or even sooner [117]. If we ignore Anthracoptilidae, the Jurassic diversification of crown-mantises recovered here is congruent with the palaeontological dating of the oldest fossil record [87].

Even though stem-mantises would date back to the Late Carboniferous, our results suggest that crown-mantis diversification would have occurred much more recently, in the Early-Middle Jurassic. This pattern of old origin and much more recent diversification is puzzling but it could be related to their major life history trait: carnivory. Before the Jurassic, there were several carnivorous lineages, including several polyneopterans. Among these lineages, Titanoptera is extinct, only known from the Triassic. Mantophasmatodea was present (and may have flourished) before the diversification of the crown-mantises [118]. Some Palaeozoic and Early Mesozoic “roachoids” (Raphidiomimoidea) were also probably carnivorous. Our dating estimates suggest that the crown-mantises would have postdated all these polyneopteran carnivores, a scenario of ‘ecological succession’ already hypothesized by Gorochov [119]. Given the controversial nature of anthracoptilids around both their taxonomic affinity and their possession of raptorial forelegs [56], we cannot confirm whether raptorial legs were already present in stem-mantis lineages or if it is a crown-mantis apomorphy that would have been a key acquisition for their diversification, after competitors had disappeared.

### Cockroaches, “roachoids” and the putative sister-taxon of Dictyoptera

Numerous and diverse “roachoid” fossils, with or without any external ovipositor, are known from at least the Westphalian (~315 Mya–Carboniferous; [120]) to the Early Cretaceous periods (~130 Mya; [87]). But their taxonomy and phylogenetic affinities to extent Dictyoptera is ambiguous [23,49]. As for modern cockroaches, all of which lack external ovipositors, their oldest fossils date back to the Early Cretaceous period (~ 120 Mya; [87,121,122]). Modern cockroaches are hence thought to have their origin in the Jurassic [87] but previous dating estimates suggest a much broader range (see for example [21,86]).

We postulate here an origin of crown-Dictyoptera in the Late Carboniferous or Early Permian (293.7–315.1 Mya), which is older than commonly thought (e.g., ~ 200 Mya in [87,117]), if one does not consider the disputed “roachoids” and Anthracoptilidae that we have discussed above. This age is congruent with the presence of “blattoid” ootheca in the Late Carboniferous, suggesting that Dictyoptera with reduced ovipositors were already present at that time, coexisting with “roachoids” with long external ovipositors [123]. This result, however, is much younger than some “roachoid” fossils and clearly invalidates the hypothesis suggesting that winged “blattoids” would date back to the Devonian [124]. We thus can reduce the chronological window associated to Dictyoptera diversification according to fossils of disputed taxonomic attribution. Nevertheless, some old “roachoid” fossils remain compatible with our dating estimates and would deserve further investigation before any conclusions are drawn about their phylogenetic affinities. Hopefully, new specimens and modern tools may help revealing new characters and assessing intra- and inter-specific variation in wing venation for more accurate interpretations [125]. They could allow assessing what are the relationships between these “roachoids” of the stem-Dictyoptera and the crown Blattodea and/or crown Mantodea.

We estimated that stem-Dictyoptera dated back to the Middle-Late Devonian (~375 Mya). This estimate is congruent with datings provided in a large-scale phylogenomic study ([86], but see [117]). It is also congruent with the hypothesis suggesting that Paoliida, a Palaeozoic insect group, would be sister-group to Dictyoptera [50].

### Origin of termites and the evolution of eusociality

There is a debate around the origin of termites, which are classically thought to date back to the Late Jurassic (150–160 Mya; [26,87,126]) but Hasiotis and Dubiel [66], on the basis of putative termite nest evidence, hypothesized that they would date back, at least, to the Late Triassic (~215 Mya). Given both the pivotal role of termites in contemporary warm ecosystems and their eusocial system, this debate has important consequences on our understanding of insect evolution.

Apart from Bourguignon et al. and Misof et al. ([26,117]; 136–170 Mya, ~130–145 Mya, respectively), recent molecular studies reported a much older and wider range for crown-Isoptera than ours (140–480 Mya in [21]; 180–230 Mya in [127]), and, even though Davis et al. [21] acknowledged that their oldest estimates are artifactual, they do not deny a possible Late Triassic, or even older, origin for termites. Here, we hypothesized a stem-termite origin in the Early Jurassic (i.e. ~ 195 Mya) and a crown diversification in the Late Jurassic (~ 150 Mya). Thus, the Late Triassic termite nest ‘evidence’ (~215 Mya; [66]) is not corroborated by our analyses, which supports previous criticisms of this fossil interpretation [62,68]. The discovery of a nest-like structure in Triassic rocks, even if similar to termite nests, could have been built by other organisms. Furthermore, the age of the embedding rocks could be different from the age of the nest itself. The fossil Stephanotermopsis rodendorfi [128], a putative stem-termite that dates back to ~290 Mya but has never been revised since its original description, does not fit either with our dating estimates or those of Ware et al. [127] or Bourguignon et al. [26]. Stephanotermopsis rodendorfi possesses some Dictyoptera-like attributes (e.g., forewing with ScP anteriorly pectinate, both RA and RP branched) but it is likely not a stem-Isoptera because it lacks any of Isoptera synapomorphy (e.g., S. rodendorfi has a long and branched Sc, a branched RA and a well individualized RP; A. Nel, pers. obs.)

Our dating estimates put into perspective the fact that termites could have been the first extant insect lineage that has evolved eusociality [129,130]. Given the difficulties associated with fossil nests, other evidence of sociality should be looked for to refine our understanding on the origin of eusociality. Among these evidences are sterile castes but fossils of these castes are rare, and the oldest one dates back to the Early Cretaceous [131]. For reproductive individuals, which are the most abundant in the oldest fossil records, only a phylogenetic position hypothesis would allow inferring sociality. Vršanský [132] suggested that the presence of a basal suture in adult termite wings could also be indicative of eusociality but this morphological character is rather associated to a life in endogean habitats and not characteristic of eusociality.

Finally, given both the recent origin of termites and their crucial role as decomposers, one can wonder how warm ecosystems functioned in the Triassic or before (see for example [133]). Myriapoda, Blattodea and some Orthoptera could also have acted as decomposers, but further evidence is needed. Thus, it remains an open question and it is worth noticing that similar questions exist for scavenger and coprophagous guilds, as these guilds are mainly composed of two recent lineages: Diptera and Coleoptera since the Cenozoic.

## Conclusions

We used a conservative approach allowing old age estimates with verified fossils and a large phylogenetic sample to elucidate the timing of diversification of Dictyoptera and of its three sub-orders (i.e. Blattodea, Mantodea and Isoptera). We provided age estimates that clarified the debates around the origin of each crown-dictyopteran group. Cockroaches and praying mantises appear as ancient lineages as assumed by some early authors, but the real situation is not so simple. The most ancient presumptive fossils of these groups were not necessarily correctly attributed and debates are far from over. In contrast, termites appear more recent than some authors suggested. As a whole, our approach showed that presumptive old ages were not all confirmed in spite of a conservative root calibration.

This chronological framework has three main evolutionary consequences. First, the pattern of old origin and much more recent diversification of praying mantises suggests a scenario of ecological succession in the major carnivorous lineages of polyneopterous insects. Second, the recent detritivory in termites could have complemented the more ancient detritivory of cockroaches. Third, we refine the chronological window during which termites evolved eusociality, potentially the first insect group to do so.

This new timescale for Dictyoptera provides an opportunity for directing future research both in molecular phylogenetics and in palaeontology. It would be necessary to investigate further the phylogenetic relationships of cockroaches, which seem more obscure than those of praying mantises and termites, and investigate undersampled families and surprisingly long branches. Blattidae, Corydiidae and Anaplectinae should be the first target in future phylogenetic studies. Also, it would be necessary to search for fossils of each group at some critical periods shown by the present dating where their occurrence is still disputable: praying mantises in the Permian and Triassic periods; cockroaches in the Permian; and termites in the Jurassic.

## Supporting Information

S1 Dataset Phylogenetic matrix. Alignment of the six molecular markers for the 793 species in nexus format.
(NEX)

S1 Methods R script. Script used to remove taxa with very short branches in each tree used for dating analyses.
(R) Click here for additional data file.

S2 Methods Pruned taxa. Tab-delimited text with the list of taxa to be pruned for each tree in dating analyses.
(TXT)

S1 Table Taxonomic sample. Table with a list of the specimens used in this study and their GenBank accession numbers for the six loci. Sequences obtained for this study are in bold.
(XLS)

S2 Table Preliminary datings. Table with the results of the different dating estimates under different smoothing values for the five main nodes (root, crown-Dictyoptera, crown-mantises, crown-cockroaches and crown-termites).
(DOC)
