# Supplementary material for: Phylogeny of Dictyoptera: Dating the Origin of Cockroaches, Praying Mantises and Termites with Molecular Data and Controlled Fossil Evidence
Source: PLoS One. 2015 Jul 22;10(7):e0130127. doi: 10.1371/journal.pone.0130127 (PMC4511787; doi:10.1371/journal.pone.0130127)
Supplement: S2 Table — Table with the results of the different dating estimates under different smoothing values for the five main nodes (root, crown-Dictyoptera, crown-mantises, crown-cockroaches and crown-termites). (DOC) [file pone.0130127.s005.doc]

**S2 Table.** **Preliminary datings (Legendre et al., 2015).** Table with the results of the different dating estimates (in million years) under different smoothing values for the five main nodes (root, crown-Dictyoptera, crown-mantises, crown-cockroaches and crown-termites).

|  | **Smoothing values** | | | | | | | | | | Maximal difference |
| --- | --- | --- | --- | --- | --- | --- | --- | --- | --- | --- | --- |
| **Nodes** | 1 | 10 | 35 | 50 | 80 | 125 | 200 | 400 | 900 | 2000 |
| Root | 470.00 | 470.00 | 470.00 | 470.00 | 470.00 | 470.00 | 470.00 | 470.00 | 470.00 | 470.00 | 0.00 |
| Dictyoptera | 310.83 | 310.53 | 309.85 | 309.50 | 308.91 | 308.22 | 307.42 | 306.27 | 305.05 | 303.33 | 7.50 |
| Mantids | 204.66 | 204.01 | 202.44 | 201.62 | 200.21 | 198.51 | 196.42 | 193.07 | 188.77 | 182.68 | 21.98 |
| Cockroaches | 284.30 | 283.95 | 283.15 | 282.74 | 282.04 | 281.22 | 280.27 | 278.91 | 277.55 | 275.95 | 8.35 |
| Termites | 169.94 | 169.81 | 169.50 | 169.34 | 169.08 | 168.77 | 168.40 | 167.86 | 167.20 | 166.13 | 3.81 |
